# Supplementary material for: Content-rich biological network constructed by mining PubMed abstracts
Source: BMC Bioinformatics. 2004 Oct 8;5:147. doi: 10.1186/1471-2105-5-147 (PMC528731; doi:10.1186/1471-2105-5-147)
Supplement: Additional File 2 — The original results of the above study (non-essential files are deleted to keep the file size under the limit set by BMC bioinformatics). [file 1471-2105-5-147-S2.bz2 › chilibotAdditionalFile2/dip05/464ID11259614E10491/html/CXCR3_CXCL9.html]

 


 **CXCR3** and **CXCL9** 
  
Found 192 abstracts in PubMed, retrieved 05.  
 

 What does Google say? 
 PDF only 
| .edu only 

---

**Interactive relationship** (e.g. stimulation, inhibition, etc)

**Neutral relationship**- AIM To assess the immunological role of human milk by analysing the concentrations of interferon gamma inducible protein of 10 kda IP 10  [ **CXCR3** ]  and monokine induced by interferon gamma MIG  [ **CXCL9** ]  in human milk from mothers of preterm and term infants.  Ref: 12856973 Acta Paediatr, 2003
- Interferon gamma inducible protein 10 IP10  [ **CXCR3** ]  and monokine induced by interferon gamma MIG  [ **CXCL9** ]  positive H& ; RS cells were detected in all 10 HL MC but only in 5 of 10 cases of HL NS and 2 of 9 cases of HL like ATLL.  Ref: 12866030 Int J CancerInt J Cancer, 2003
- The chemokine receptor  **CXCR3**  is predominantly expressed on T lymphocytes, and its agonists  **CXCL9** , CXCL10 and CXCL11 are IFN gamma inducible chemokines that promote Th1 responses.  Ref: 12884299 Eur J Immunol, 2003
- DESIGN AND PATIENTS Immunocytochemistry and reverse transcriptase polymerase chain reaction RT PCR amplification were used to analyse the expression of monocyte chemoattractant protein MCP 1, RANTES regulated upon activation, normal T cell expressed and secreted, macrophage inflammatory protein MIP 1alpha, MIP 1beta, interferon IFN gamma inducible protein IP 10  [ **CXCR3** ]  and monokine induced by IFN gamma Mig  [ **CXCL9** ]  in thyroid tissue from patients with Hashimoto s thyroiditis n = 4, Graves disease n = 6 and nonautoimmune multinodular goitre n = 4.  Ref: 12864798 Clin Endocrinol (Oxf), 2003

**Non-interactive relationship** (e.g. studied together, co-existance, homology, etc.)

- RESULTS Significant amounts of IP 10  [ **CXCR3** ]  and MIG  [ **CXCL9** ]  were detected in human milk.  Ref: 12856973 Acta Paediatr, 2003
- CONCLUSION We postulate that TFC may play a role in the pathogenesis of autoimmune thyroid disease as they are able to express the chemokines MIP 1alpha, MIP 1beta, MCP 1, RANTES, IP 10  [ **CXCR3** ]  and Mig  [ **CXCL9** ]  that would promote the infiltration of immune cells into the thyroid gland.  Ref: 12864798 Clin Endocrinol (Oxf), 2003
- RESULTS Expression of MCP 1, RANTES, MIP 1alpha, MIP 1beta, IP 10  [ **CXCR3** ]  and Mig  [ **CXCL9** ]  was demonstrated in all Hashimoto s and most Graves thyroid specimens but very little expression was detected in the nonautoimmune goitre samples.  Ref: 12864798 Clin Endocrinol (Oxf), 2003
- IP 10  [ **CXCR3** ]  and MIG  [ **CXCL9** ]  mRNA expression levels in cellular components of human milk were determined by RT PCR.  Ref: 12856973 Acta Paediatr, 2003
- METHODS IP 10  [ **CXCR3** ]  and MIG  [ **CXCL9** ]  levels of colostrum, early milk, mature milk and sera were measured by enzyme linked immunosorbent assay ELISA.  Ref: 12856973 Acta Paediatr, 2003
- High levels of RANTES CCL5, MCP 1 CCL2, IP 10  [ **CXCR3** ]  CXCL10, and Mig  [ **CXCL9** ]   **CXCL9**  simultaneously with mononuclear cell infiltration in the lungs was found.  Ref: 12875978 Am J Pathol, 2003
- Expression of IP 10  [ **CXCR3** ]  and MIG  [ **CXCL9** ]  genes and proteins in the milk cells as well as in mammary gland epithelial tissues was detected by RT PCR and immunohistochemistry.  Ref: 12856973 Acta Paediatr, 2003
- The concentrations of IP 10  [ **CXCR3** ]  and MIG  [ **CXCL9** ]  in colostrum and early milk were significantly higher than those of sera from healthy controls or lactating mothers.  Ref: 12856973 Acta Paediatr, 2003
- There were significant correlations between MIG  [ **CXCL9** ]  and interferon gamma IFN gamma or IP 10  [ **CXCR3** ]  levels p <.  Ref: 12856973 Acta Paediatr, 2003
- CCR3 functional responses are regulated by both  **CXCR3**  and its ligands  **CXCL9** , CXCL10 and CXCL11.  Ref: 12884299 Eur J Immunol, 2003
- CONCLUSION IP 10  [ **CXCR3** ]  and MIG  [ **CXCL9** ]  in human milk, probably derived from milk cells and mammary gland epithelial cells, may contribute to the migration and activation of intestinal T lymphocytes to enhance mucosal immunity during the early neonatal period.  Ref: 12856973 Acta Paediatr, 2003
- IP 10  [ **CXCR3** ]  and MIG  [ **CXCL9** ]  protein expression in mammary gland tissues was analysed by immunohistochemistry.  Ref: 12856973 Acta Paediatr, 2003
- However, 2 of 5 cases of HL like ATLL with EBV infection and 2 of 2 HL NS with EBV had IP10  [ **CXCR3** ]  MIG  [ **CXCL9** ]  positive H& ; RS cells.  Ref: 12866030 Int J CancerInt J Cancer, 2003
